# Manufacture of Low-Na White Soft Brined Cheese: Effect of NaCl Substitution with a Combination of Na-K Salts on Proximate Composition, Mineral Content, Microstructure, and Sensory Acceptance

**DOI:** 10.3390/foods13091381

**Published:** 2024-04-30

**Authors:** Vladimir S. Kurćubić, Steva Lević, Vlada Pavlović, Ružica Mihailović, Aleksandra Nikolić, Mirjana Lukić, Jelena Jovanović, Bojana Danilović, Mira Milinković, Fatih Oz, Volker Heinz, Igor Tomasevic

**Affiliations:** 1 Faculty of Agronomy, Department of Food Technology, University of Kragujevac, Cara Dušana 34, 32000 Čačak, Serbia; 2 Faculty of Agriculture, University of Belgrade, Nemanjina 6, 11080 Belgrade, Serbia; slevic@agrif.bg.ac.rs (S.L.); vladimirboskopavlovic@gmail.com (V.P.); 3 Veterinary Specialist Institute Kraljevo, Žička 34, 36000 Kraljevo, Serbia; pavicruzica@yahoo.com; 4 Department of Sensory and Physical Testing with Parasitology, Institute for Meat Hygiene and Technology, Kaćanskog 13, 11000 Belgrade, Serbia; aleksandra.nikolic@inmes.rs (A.N.); mirjana.lukic@inmes.rs (M.L.); jelena.jovanovic@inmes.rs (J.J.); 5 Faculty of Technology, University of Niš, Bulevar Oslobođenja 124, 16000 Leskovac, Serbia; bojana.danilovic@tf.ni.ac.rs; 6 Institute of Land, Teodora Drajzera 7, 11000 Belgrade, Serbia; miramilinkovic@yahoo.com; 7 Faculty of Agriculture, Ataturk University, Erzurum 25240, Türkiye; fatihoz@atauni.edu.tr; 8 DIL German Institute of Food Technology, Prof.-von-Klitzing-Str. 7, D-49610 Quakenbrück, Germany; v.heinz@dil-ev.de

**Keywords:** white soft-brined cheese (WSBC), Na reduction, NaCl substitution, sensory evaluation, microstructure of WSBC

## Abstract

All over the world, especially in Western societies, table salt intake that is inordinately higher than the acceptable level has been observed. An excess of Na in the human diet, mostly from processed foods, is becoming the “number one killer”, leading to increased blood pressure. Therefore, the food industry is faced with a need to reduce Na in human nutrition in an effort to raise public health protection to a higher level. In this study, a commercially available combination of Na/K salts (COMB) at different concentrations was used as a NaCl substitute in the production of a modified, healthier, Na-reduced cheese. Samples of the modified low-Na white soft-brined cheese (WSBC) were produced by adding four different concentrations of COMB to production lots PL-1 to PL-4, and the control (CON) samples were prepared by salting with the usual, non-reduced concentration of NaCl. The effects of NaCl replacement on the physical–chemical parameters, major- and micro-elements, and microstructural and sensory properties of the WSBC were investigated. The obtained results indicated that there was no significant influence on the ash content, pH, and a_w_. The Na and K levels differed among treatments (*p* < 0.001). The lowest Na level in this study was recorded in PL-4 (only COMB was added) and was 334.80 ± 24.60 mg/100 g. According to the Na content, WSBC PL4 can be labeled with the nutrient claim “reduced amount of Na”. A significant difference (*p* < 0.05) was noticed in overall acceptance between the CON and PL-4, with no statistically significant difference found amongst other WSBC production lots. The replacement of NaCl resulted in a slightly greater firmness of the WSBC. The results confirm the possibility of producing low-Na WSBC when optimal amounts of a suitable mineral salt are used as a substitute for NaCl, thus reducing the risk of high Na intake in the human body through the consumption of evaluated cheese.

## 1. Introduction

In modern society, sodium (Na) intake is often uncontrollably higher than the scientifically established, recommended, and acceptable daily intake, which dramatically increases the risk of the occurrence of arterial hypertension, cardiovascular disease (CVD), and stomach cancer [1,2]. Limiting dietary sodium intake is the most important procedure in the strategy of preserving public health by preventing the occurrence of CVD and chronic kidney disease in the human population [3]. The essential importance of reducing Na content in the human diet was impressively described by Cappuccio et al. [4] in their inspiring review “Sodium and Health: Old Myths and Controversy Based on Denial”.

The current situation of poor nutritional and medical knowledge in the wider population, lack of information about World Health Organization (WHO) recommendations, and modern lifestyles (unhealthy eating habits: fast food, processed food, restaurant food) suggests that table salt, particularly its uncontrolled and careless use, can damage body functions and human health. WHO recommendations on reducing the content of Na in food have been adopted and related national strategies have been implemented by 75 countries around the world [5,6,7].

For adults, the WHO recommends a maximum Na intake of less than 2000 mg daily (<5 g NaCl daily) [8]. The first Global Report on Na intake reduction was published in 2023 and evaluated the achievement of the strategic goals of reducing Na content in food within member states (out of 194 countries, 79% have a policy dedicated to reducing Na). The creation of the Global Report was made possible by using multiple data sources and models to determine the level of Na intake in the human diet, the implementation of national policies, and the corresponding score, as well as the impact of policy on Na intake and CVD [8].

Processed foods (including cheeses as extremely attractive dairy products to consumers) are estimated to account for over 70% of dietary Na intake (depending on cheese variety), which is five times higher than known levels in natural raw materials [9,10,11]. Cheese is recognized as a food with a high Na content (in the range of 0.5% to 2.5% NaCl for cheeses of different hardness). Blue-type cheeses contain 3 to 5% NaCl. White soft-brined cheese (WSBC) can contain up to 7% NaCl; thus, low-Na cheese is necessary for the implementation of public health improvement strategies [7]. Forty grams of cheese with 2% NaCl corresponds to 16% of the daily salt intake as recommended by the WHO [12].

Such an alarmingly high salt/Na content in cheeses requires technological reformulation, but the reduction in saltiness is a risk to customer sensory satisfaction. In a survey of Serbian consumers’ attitudes regarding the saltiness of local traditional cheeses, only consumers over 55 rated them as too salty [13]. An alternative strategy can be seen in the production of cheese subtypes with low levels of Na compared to existing cheeses. Healthier and more functional added-value dairy products are intended to meet the demands of particular groups of more conscious or demanding customers [14,15]. Salt/Na reduction programs must involve a multi-segmented, multidisciplinary approach with a reformulation of cheeses and other foods of animal origin, clear salt content labeling, and public education campaigns with as wide a reach as possible so that well-informed consumers make correct decisions when buying food [13]. Recent research has indicated that more consumers want to buy healthier cheeses with nutritional information about reduced table salt levels [16].

Sodium chloride has significant techno-functional effects on the rheological properties, texture, and safety of cheeses. It (a) modifies the physico-chemical characteristics of whey and cheese rind. The most important thing is to keep the value of water activity (a_w_) at a low level; (b) increases the water binding capacity; (c) controls the growth of microbiota necessary for optimal cheese ripening; (d) creates optimal conditions for enzyme action and biochemical changes during cheese ripening; (e) inhibits the development of pathogens or bacterial spoilage agents; (f) regulates enzyme activity for the breakdown of proteins (predominantly casein) and fats, which leads to the creation of various compounds (flavor compounds, free amino acids, peptides, and free fatty acids); and (g) acts as a flavor enhancer [14,17,18].

A capital goal for public health authorities is Na reduction in the human diet, and the need for food reformulation is a significant and often enigmatic challenge for the food industry—evolution in food processing [19]. The planned strategies can be realized in two ways, by reducing the level of table salt or by its partial substitution with other salts. A large number of studies [18,20,21,22,23,24,25,26,27,28] have been carried out to evaluate the effect of NaCl reduction on the quality (physical-chemical and sensory properties) of various cheeses or meat products. If Na content in cheese is reduced by 15%, consumer acceptance will not change [16].

According to nutrition claims, products are classified as follows: (a) without Na (less than 5 mg per labeled portion), (b) with a low Na content (≤140 mg per labeled portion), or (c) with a reduced Na content (at least 25% less Na than the corresponding reference food). Such nutrition claims positively correlate with increased consumer willingness to purchase cheeses with a reduced Na content [16]. Recent studies have shown research success in formulating products with a low Na content by moderately replacing NaCl with KCl. This approach can lead to reductions in Na levels of 13%, 25%, and 67% in mozzarella, cheddar, and processed cheese, respectively, with a slight deterioration in sensory attributes, whereas greater reductions can lead to the usual unpleasant metallic aftertaste and bitterness when using KCl as a replacement for NaCl [29].

Intensive research should lead to the implementation of strategies to reduce the content of salt/Na in processed products in the food industry, with the ultimate goal of decision-makers reaching a clear and explicit salt reduction attitude, leading to the adoption of mandatory regulations. Those entities in the food business that do not meet the requirements will find it difficult to operate and their rating on the market will decrease.

Sodium reduction in foods can be obtained by dissimilar procedures: (1) reduced addition of table salt; (2) substitution of the desired proportion of NaCl with different available salts [24,27,30,31]; (3) astute application of masking and taste-enhancing agents [32]; (4) a combination of the above-mentioned approaches [33]; (5) addition of plant extracts, lyophilizates, and essential oils, as well as spice extracts, to products of animal and plant origin [25,27,34,35,36,37]; (6) application of innovative and optimized physical forms of salt [38,39], and (7) alternative contemporary processing methods [40]. All these methods are based on the following three principles: (1) manipulation of the sense of taste with a pulsating salt concentration or a multisensory combination; (2) control of the Na concentration (depending on the time needed for Na to reach the taste receptors), and (3) replacement of Na with other applicable salts and additives [41].

The most common procedure for reducing Na content in different varieties of cheese is to replace a proportion of NaCl with KCl, as described in white soft-brined cheese (WSBC) from Serbia [18], Portuguese Sao Joao [42], Feta [43], and Turkish White Cheese [44,45]. A combination of Na-K salts has been successfully applied in different types of cheese without any deterioration in cheese quality: Halloumi [46], Kefalograviera [47], White [48], imitation cheese [49], Feta [42,50], fresh Minas [51], and Iranian White cheese [52]. The most extensive production and consumption of WSBC is in Serbia, where it is generally characterized by a high NaCl concentration (5.5–9.0%) [13,53,54,55]. The fact that WSBC is by far the most massively produced and most commonly consumed cheese in Serbia indicates that reducing the Na content in this cheese is extremely important for the improvement of public health. The primary goal of this research was to characterize the physical-chemical and sensory attributes of WSBC, as well as to determine the potential change in cheese microstructure caused by NaCl replacement. This study is one of the pioneering works on replacing NaCl with various concentrations of the commercially available NaCl/KCl mineral salt combination in Serbian WSBC.

## 2. Material and Methods

### 2.1. White Soft-Brined Cheese (WSBC) Formulation, Processing, and Sampling

Five different production lots of WSBC were prepared (control—CON and 4 experimental lots—PL-1, PL-2, PL-3, and PL-4), each weighing 5 kg (Table 1). All 5 variants were made from previously partially skimmed milk (by “removing” the rind of the cream, the so-called kaymak). Kaymak is formed primarily by fats, which float out of the milk in the rest phase in shallow open containers, and a small amount of protein creates a matrix in which the fats are embedded. All 5 varieties of WSBC samples were obtained by the same production process, up to the salting stage.

The milk received from “Gaj” Dairy Ltd. (Čačak, Western Serbia) was thermally processed by being passed over the plates of the pasteurizer (Milk Pasteurizer C. 4580; manufacturer: “RIB JOVANOVIĆ”, Kruševac, Serbia; type: with 4 sections), with a zigzag relief, by high-temperature pasteurization at a temperature of 92 to 95 °C for a duration of 1–5 s. After successful heat treatment, the milk was poured into hygienically prepared pans (detergent-washed, rinsed, disinfected, then rinsed), where it stood overnight, after which it was skimmed by collecting the cream. The skimmed milk was again delivered to the pasteurizer through a system of marked pipelines, using pumps. The second pasteurization was carried out by a short-term pasteurization process, in the temperature range of 72 to 75 °C, for 15–20 s, after which the pasteurized milk was sub-cured as the first stage in cheese production.

After pasteurization, the milk was pumped from the pasteurizer to cheese vats (milk curdling vats, “RIB JOVANOVIĆ; Kruševac; type: KD-63,5). The temperature of the milk in the cheese vats during cheesemaking was 43 °C (vat capacity was 2000 L of milk). Curdling was done using rennet (“Sirela—Maja”—liquid rennet of microbiological origin based on chymosin obtained from the fungi *Rhizomucor miehei* and *Mucor miehei,* rennet strength 1:5000 i.e., 1 mL of rennet is coagulated 5000 mL of milk) at a concentration of 0.2% (MTM Sirela d.o.o. Čačak, Kostićevina 2 No. 57, 32212 Preljina, Čačak, Serbia) and calcium chloride (CaCl_2_) at a concentration of 10–20 g/100 L of milk, both from the same manufacturer. The milk curdling and curd formation phase lasted about 1 h. Thereafter, the second stage of cheese production involved processing (cutting) the curd into small cubes, 0.5–1 cm in size, with dairy equipment for curd cutting (lyres and harps), in order to encourage the separation, drainage, and expulsion of whey (*syneresis*).

The third and fourth stages (molding with pressing) began with the transfer of cheese grains into molds and onto the cheese tables equipped with strainers. When the molds were filled, the curds were pressed for 1.5–2 h, which had an additional syneretic effect. The whey was collected in a special chrome tub, where it was then cooled to a temperature of 5 °C and later used for brining the cheese.

After pressing, the WSBC was cut, using cheese knives, into cubes (slices) of certain dimensions (according to the consumers’ habits). Then, the WSBC was salted using a dry process.

Cheese samples originating from the control (unmodified, CON) group were salted with 2% table salt (iodized table salt, Solana d.d. Tuzla, Ulica Soli no. 3, 75 000 Tuzla, Bosnia and Herzegovina), and produced according to the original manufacturer’s recipe. WSBC samples from the 4 mentioned production lots (PL) were salted with a mixture of Na/K salts in different proportions.

In order to examine the possibility of replacing Na salt (NaCl) with the Na-K salt combination (hereinafter: COMB), five experimental groups were formed. The combinations of NaCl and COMB (with different ratios used for salting the experimental cheese samples), as well as the sample codes used in the presentation and discussion of the test results, are given in Table 1.

The (K-Na) salt combination with a reduced Na content was used for salting WSBC samples and preparing whey (brine) for soaking cheese: Na max 27 g/100 g, K min 16 g/100 g, iodine 12–18 mg/kg, anti-caking agent E536 max 10 mg/kg; produced and packaged by: KRISTAL SO d.o.o. Belgrade, packaging of 350 g; date of iodization and packaging: 16/11/2021; best used by: 10/12.2024).

After salting, the WSBC cubes were packed in appropriate packaging (marked PVC square containers that seal well) and soaked with the prepared cooled brine (collected whey with added salt, NaCl was added to the control and PL-1–PL-3 WSCB samples, whereas COMB was used for PL1–4 samples).

The prepared samples were left to ripen in a cold chamber, at a constant temperature of 4 °C, for about two weeks, until maturity was reached (According to the Rulebook on the Quality of Milk Products and Starter Cultures, Official Gazette of the RS Issue No. 34/2014, cheese is considered mature after 7 days of ripening and ripening can last 30 or more days until commercial maturity).

### 2.2. Proximate Composition of Reformulated White Soft Brined Cheeses (WSBC)

Before the homogenization of the experimental cheese samples, the WSBC slices were removed from the brine and drained for 2 min. All slices were ground and homogenized in order to obtain uniformity before physical and chemical analyses. The proximate composition of reformulated WSBC including moisture content, dry matter, protein, fat, ash, titratable acidity, chloride level, pH, and a_w_ was determined following the AOAC (1990) standardized methods [56,57,58].

### 2.3. Macroelements and Microelements

Before determining the content of macroelements, a porcelain pot was preheated for at least 120 min in a muffle furnace set to 550 ± 20 °C, and then cooled in a desiccator and weighed. The tested homogenized sample of cheese (about 5 g) was placed in a measuring pot and progressively heated on a hot plate until the sample was carbonized.

The ash was dissolved by heating with 5 mL of hydrochloric acid and 5 mL of deionized water, and the solution was transferred quantitatively to a 100 mL volumetric flask.

The contents of macroelements (Na, K, Mg, and Ca) and microelements (Fe, Cu, and Zn) were determined by PinAAcle 900T AAS (PerkinElmer, Inc., Waltham, MA, USA). The calibration curves were constructed by diluting the Fisher Chemical standard solution for Na, K, Mg, Ca, Fe, Cu, and Zn.

The content of P was determined by a UV–VIS 1800 spectrophotometer (Shimadzu, Tokyo, Japan).

All measurements were performed in triplicate at a minimum, and the results were presented as mean values.

### 2.4. Microstructure Analysis (Scanning Electronic Microscopy (SEM)) of Reformulated White Soft-Brined Cheeses (WSBCs)

Scanning electron microscopy (SEM) was used to analyze the microstructure of cheeses. Fresh cheese samples were reduced to pieces ~ 5 × 5 × 15 mm, followed by the preparation procedure described by Kuo and Gunasekaran (2009) [59]. Dehydrated samples were stored in absolute ethanol until critical point drying with carbon dioxide. The drying procedure was performed using a K850 Critical Point Dryer (Quorum Technologies, Laughton, Lewes, UK). Dried cheese samples were placed on stubs and sputter-coated with gold using a Sputter Coater SCD 005 (BAL-TEC AG, Balzers, Liechtenstein). SEM analysis was performed by a JEOL JSM 6390LV scanning electron microscope (JEOL, Tokyo, Japan).

### 2.5. Sensory Acceptance of Modified Low-Na WSBC and Control Samples

Sensory testing was conducted after 14 days of cheese ripening. A panel consisting of five trained assessors of different ages performed the sensory evaluation. Assessors were previously tested for detecting and recognizing various tastes [60], odors [61], and colors [62]. The sensory evaluation of the cheeses was carried out using the quantitative descriptive test [63].

Sensory acceptance was assessed by evaluating five selected characteristics: color, oral texture, odor (orthonasal olfaction), taste, and overall acceptance. The selected sensory characteristics were rated using a numerical descriptive scale, with a grading scale from 1 to 5 (1—unacceptable, 5—extremely acceptable), divided by 0.5 and resulting in a scale with 9 alternative responses.

Cheese evaluations were conducted in sensory booths, designed according to the SRPS ISO 8589:2015 standard [64], under white light, at the Sensory Laboratory of the Institute of Meat Hygiene and Technology in Belgrade, Serbia. Before the sensory analysis, one block of five cheese variants (CON, PL-1, PL-2, PL-3, and PL-4) was taken out of the brine, cut into 2.5 cm^3^ cubes, and served at room temperature on white, plastic plates. The samples were encoded with three-digit codes and presented to the assessors in a random order. Sensory evaluation was carried out in an air-conditioned room (20 °C) with a humidity level between 50% and 70%. Assessors were provided with mineral water and bread to cleanse their palate between samples.

Sensory acceptance results were the mean value ± standard deviations given by the five assessors.

### 2.6. Statistical Analysis

The results of the proximate composition of WSBC were subjected to one-way ANOVA. Recipe formulation was assigned to the main effect and Tukey’s HSD test was used to identify significant (*p* < 0.05) differences between means. Statistical analyses were performed by the software Statistica 12.5 (StatSoft, Inc., Tulsa, OK, USA) and presented as a mean ± standard deviation (SD).

The statistical analysis of the results of the cheese samples’ sensory evaluation was conducted using Minitab Inc., State College, PA, USA, ver. 17. The results were expressed as the mean value and standard deviation and were calculated using column statistics on 10 values for each examined parameter (each parameter was rated twice by each of the five assessors). Significant differences in the mean values of the examined parameters between experimental and control groups were determined using one-way ANOVA analysis and by the Tukey comparative test. Differences were considered significant at *p* < 0.05.

## 3. Results and Discussion

### 3.1. Proximate Composition of Modified Reduced-Na WSBC

The effect of using different concentrations of Na/K mineral salt combination replacements (COMB) on moisture content, dry matter, protein, fat, ash, titratable acidity, chloride level, pH, and a_w_ is presented in Table 2. The proximate composition of WSBC in terms of moisture, dry matter, protein, fat, and chloride content was significantly (*p* < 0.05) affected by the substitution of NaCl with KCl between specific production lots (PL) (Table 2). No significant differences were observed between the WSBC control and experimental samples for ash content and physical parameters (pH and a_w_). The results obtained from this study were similar to those of the other authors who examined the quality of reduced-salt cheese [18,45,65,66,67,68,69,70,71].

A significant number of scientific tests have pointed to the non-significant effect of partial NaCl substitution by KCl on the moisture, fat, and protein contents of Mozzarella cheese [72], Kefalograviera cheese [47], Feta cheese [43], Halloumi cheese [46], Coalho cheese [73], and Akawi cheese [65]. Conflicting results have also been published [74,75]. Significant variations (*p* < 0.05) were reported for the proximate composition of Cheddar cheeses made by the replacement of NaCl with KCl. The results obtained from this study were similar to those of other authors who examined the quality of salt-reduced cheese [18,45,65,66,67,68,69,70,71], and the similarity is greatest when comparing the results of testing the same parameters in testing the influence of salting with different K-Na salt mixtures in the most related types of cheese (Turkish white cheese) [45].

A decreasing trend for pH values was observed as the degree of replacement of NaCl with the COMB mixture increased, without statistically significant differences, and the pH values were within the limits for WSBC (Table 2). Contrary to the results obtained in this study, the addition of different substitutes for NaCl in Turkish white cheeses in order to reduce the Na content in them led to an increase in the pH value of the cheeses compared to the unmodified control samples [45]. Similar research on Cheddar, Mozzarella, Halloumi, Akavi, Nabulsi, Feta, and Kefalograviera cheese samples contradicted the study [45] because their results indicated that similar pH values were observed in samples of cheeses salted with different mixtures of NaCl and KCl compared to control samples (that is, Na content in cheese samples did not significantly affect their pH values) [47,50,65,76,77,78]. There are also opinions that other factors such as the quality of raw materials may influence the pH more than the Na content [70].

The a_w_ values of the WSBC samples from the CON and PL-1 to PL-4 production lots were not affected by Na reduction (*p* > 0.05), probably due to the impact of solute concentration and moisture content on a_w_ [53]. The values of a_w_ in our research are in accordance with the results of the other authors, with a slight decrease in the value of a_w_ as the Na content decreased in the cheese samples PL-1 to PL-4 [68,73]. Contrary to such conclusions, there are published results on significantly higher a_w_ in experimental Cheddar cheese prepared by 75% NaCl substitution with KCl [79]. Similar results were observed (increase in a_w_ value) when testing Mozzarella cheese salted with KCl as a substitute for NaCl [80]. The results obtained as part of the study presented in this paper for aw values in cheese samples from different experimental groups indicate that the values did not differ significantly, as shown by recent similar test results of NaCl reduction or partial substitution with KCl on the characteristics of soft cheeses [19].

In this study, the ash content decreased with decreasing Na content (from a maximum of 3.45% in CON to 3.13% in PL-2), i.e., it was lower in the experimental treatments, but without significant differences. In another experiment, when the KCl: NaCl ratios during the salting of three groups of test cheeses were 0:100, 25:75, and 50:50, the ash content also decreased as the Na content decreased: 3.67 ± 0.14, 3.46 ± 0.19, and 3.14 ± 0.12, respectively [66]. It was noted that the values for the ash content were very similar in both studies.

The physical-chemical analysis of fifty-four randomly collected samples of Egyptian low-salt soft cheeses (from different localities in Alexandria and the El Behera Governorate) [71] determined the respective mean values of pH (5.83 ± 0.52, 5.40 ± 0.70), acidity% (0.53 ± 0.31, 0.67 ± 0.35), moisture% (57.95 ± 3.54, 56.76 ± 4.91), fat/DM% (23.72 ± 5.76, 25.45 ± 12.99), and salt% (1.80 ± 0.49, 1.78 ± 0.62). The results of physical-chemical tests in our study deviate from the above-mentioned values, probably due to different production methods, the quality of raw materials, and the length of the ripening period. In the Egyptian study, samples of low-Na cheeses were taken randomly from the market.

The significant differences between all treatments observed in terms of moisture, dry matter, fat content, and titratable acidity did not show a regular pattern. Therefore, we assumed that the observed differences were due to variations in the cheese slices and not due to different levels of table salt substitution with the mineral salt combination substitutes (COMB).

Na and K levels differed between treatments (*p* < 0.05). Na content decreased with an increase in K concentration, which was also detected by other authors [65,67,77,81]. KCl has a reduced ionic strength compared to NaCl, which reduces protein solubility during salting, directly affecting the cheese matrix [51]. A group of authors investigated thirty samples of Minas Padrão cheese purchased at the market and found Na content between 150 and 967 mg 100 g^−1^ (594 mg 100 g^−1^ average). This value is close to the content found in the study of Felicio et al. [82] for the control treatment with 100% NaCl (without replacing Na).

According to Serbian legislation (Rulebook on Nutrition and Health Claims Labeled on Food Declaration, Official Gazette of the RS Issues Nos. 51/2018, 103/2018 and 110/2023, Annex 1. Nutritional claims and terms applicable to them) [83], four food categories with a reduced salt content are stipulated: (1) Low Na/Salt, (2) Very Low Na/Salt, (3) No Sodium or Salt-Free, and (4) No Sodium/Salt added. Based on this regulation, the examined WSBC samples in this study could hypothetically be classified into one of the first two food categories. A claim that a food is low in Na/salt, and any claim likely to have the same meaning for the consumer, may only be made if the product contains no more than 0.12 g of Na or the equivalent value for salt per 100 g or 100 mL (Na × 2.5 = salt). In the same Annex 1 of the Rulebook, the option for the claim “reduced quantity (name of nutrient)” is specified. The statement that the amount of one or more nutrients is reduced can be stated for micronutrients only if there is a difference of 10% in the reference values determined by the regulation governing the area of declaration, labeling, and advertising. The same statement can be considered acceptable if Na or the equivalent value of salt (Na × 2.5 = salt) is reduced in the food by 25%. In this study, the analysis of the WSBC made with a reduced N content clearly shows that it meets the labeling requirements for the nutrition claim, as nutrition and health claims should be based on and supported by generally accepted scientific evidence. The Ministry of Agriculture, Forestry, and Water Management or the Ministry of Health of the Republic of Serbia may require the Food Business Entity to substantiate the nutrition statement it provided when labeling the product. In Appendix 2 of the same Rulebook, entitled “List of Approved Health Claims”, under item number 54, types of health claims for Nutrient/substance/food/food category 13.1 “Food with a low or reduced Na content” are listed. The health declaration reads: “Reducing Na intake helps maintain normal blood pressure “, in accordance with the scientific opinion on the substantiation of health claims related to low-Na foods and maintenance of normal blood pressure [84].

This is beneficial because of the potential health risks of excessive Na consumption, since the ingestion of 30 g WSBC cheese accounts for 23.13% of the amount of Na consumed by one person daily (462.6 mg Na/30 g of WSBC vs. 2000 mg Na/daily). In the calculation and later comparison, we used the results of other authors on the content of macroelements (Na) in Serbian white brined cheese manufactured with different ratios of NaCl and KCl (1542.0 ± 146.0 Na mg/100 g) in the cheese variants with 100% Na after 60 days of ripening [18]. If modified cheese is to be labeled “low-Na”, cheese should contain less than 280 mg/100 g of Na [85]. The lowest Na content in this study was recorded in PL-4 (where no NaCl was added, but only a mixture of Na and K salts (COMB) was used), and it was 334.80 ± 24.60 mg/100 g of Na in WSBC samples. PL-4 samples scored the worst in the sensory evaluation of the modified WSBC. In PL-4 samples, the Na content was close to the value of the nutrient claim “low-Na”, in accordance with the legally prescribed nutrition statement. In the sensory assessment, the control cheese samples (produced by the usual procedure, without replacing NaCl with COMB) were rated higher by all five assessors, and the experimental PL-4 samples were rated the lowest, but still with a minimum rating of 3 (acceptable). This fact indicates that even the maximum Na reduction in the cheese (100% applied COMB) did not significantly affect the investigated sensory attributes of the cheese samples.

### 3.2. Contents of Major Elements (Na, K, Mg, Ca, and P) and Microelements (Fe, Cu, and Zn) in the Modified WSBC and Control Samples

The concentrations (mg/100 g) of major elements (Na, K, Mg, Ca, and P) and microelements (Fe, Cu, and Zn) in five experimental WSBCs at 14 d of ripening are presented in Table 3. The mineral content of the WSBC was correlated with the composition of the added salt mixture. All treatments significantly reduced the Na content by more than 35%. The mineral contents of WSBC samples were affected by the salt concentration. A significant (*p* < 0.05) difference in K content was observed between the samples of the control (CON) and all experimental production lots of WSBC (PL-1 to PL-4). Sodium content progressively decreased, with no significant differences between PL-1, PL-2, and PL-3. On the other hand, K content progressively and significantly increased with the increasing amount of NaCl replacement with COMB. Therefore, the salt added to the modified low-Na WSBC during production contributed to a significantly lower (*p* < 0.001) Na content in WSBC between certain production lots. As expected, a positive correlation was found between NaCl concentration and Na content in WSBC, i.e., decreasing the added NaCl concentration decreased the Na content in WSBC samples, as observed in white salted cheese [86] and Iranian white cheese [87]. In addition, partial replacement of NaCl with other salt mixtures such as KCl, CaCl_2_, and MgSO_4_ led to a decrease in the Na content of Turkish white cheese during 90 days of ripening [45]. Sodium has a crucial role in improving casein hydration by transposition with Ca or Ca-phosphate from para-casein (Na is bound to casein micelle fractions) [88].

**Figure 1:**
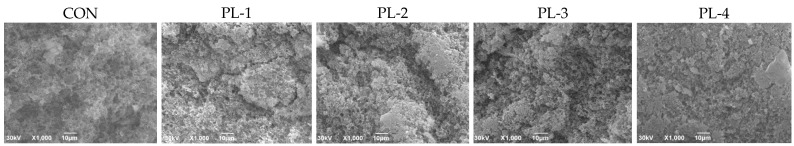
Scanning electron micrographs of Serbian white soft-brined cheese (WSBC) with a reduced Na content: Cheese code: CON—cheese with 100% NaCl and 0% added Na/K salt combination (COMB); PL-1—cheese with 75% NaCl and 25% COMB; PL-2—cheese with 50% NaCl and 50% COMB; PL-3—cheese with 25% NaCl and 75% COMB; PL-4—cheese with 0% NaCl and 100% COMB.

Calcium is one of the main minerals in cheeses and shows a number of extremely important effects: (a) enables the micellar structure of casein; (b) directly impacts the texture of cheese by building a protein 3D network (structural matrix) (c) stimulates stronger protein–protein interactions, which leads to a firmer final texture of the cheese and lower meltablity; and (d) exhibits powerful nutritional value [88]. A significant difference (*p* < 0.05) in Ca content was observed between CON–PL-2 and CON–PL-3, as well as between modified PL-1–PL-3, PL-2–PL-3, and PL-2–PL-4 of WSBC. Therefore, at the NaCl replacement level of 50% and more, a significantly higher Ca content in cheese was observed. Higher Ca content induces stronger interactions between proteins [87], which is reflected in the firmer cheese texture observed in scanning electron micrographs (Figure 1).

Phosphorus contributes significantly to the acid/base equilibrium, synthesis of nucleic acids, formation of amino acids, neural system constitution, and creation of phospholipids and high-energy bonds [89]. Research results have reported that brine concentration had no significant effect on P content in Iranian white cheese [87] because P bonded to the casein micelle fraction in its insoluble form. The contents of P determined in experimental cheeses [90] were lower than the values in Italian fresh cheeses [88] and higher than in Iranian white cheese [87] and Prato cheese [91]. A significant difference (*p* < 0.001) in P content was observed between CON–PL-2 and CON–PL-3, PL-1–PL-2, PL-1–PL-3, and PL-2–PL-4 of WSBC. With salt treatment, P content changed differently across the PLs of cheeses [45]. The results of this study are similar to the results of Akan and Kinik (2018) [45], which revealed the important differences in terms of the content of Na, K, Ca, and P among the samples of five various groups of Turkish White cheese with potentially low Na content.

In milk, zinc is linked to casein fractions and mostly remains in the whey [92]. In our study, when Na concentration decreased, the content of Zn in modified experimental cheeses showed a decrease (except in PL-4 samples, where a slight increase was recorded compared to the control samples (100% Na)). A low pH value and migration towards the soluble phase might have caused the reduction in Zn content in certain experimental groups of cheeses [90], and a similar phenomenon was described during the related examination of Prato cheeses [91].

The contents of Cu and Fe in cheese testify to the final quality of the product because these metals have nutritional and biological importance. The two metals act catalytically and promote the oxidation of lipids, resulting in the development of an unpleasant odor, by binding primarily to proteins and lipoproteins positioned in the membrane of the fat globule in milk. No significant difference (*p* > 0.05) in Mg and Fe content was observed between the control and all modified production lots (PL-1 to PL-4) of WSBC. The contents of Cu and Fe increased with increasing NaCl concentration in experimental cheeses probably under the influence of low pH values and their migration through the soluble fraction [89], and the same was recorded in this study (Table 3).

Cheese salted with a 1:1 NaCl/KCl mixture had an acceptable Na:K ratio (Na content of 165 mg per 28.48 g serving) but could not be labeled as “low-Na” cheese. For such labeling, the Na level would have to be ≤140 mg per 28.48 g. The content of K in modified cheeses (salted with a mixture of NaCl/KCl in a 3:1 or 1:1 ratio) was about six or twelve times higher than in cheese samples from the control group. Therefore, the authors concluded that Feta cheeses salted with a mixture of NaCl and KCl in the ratio 3:1 or 1:1 (*v*/*v*) instead of NaCl alone are of very acceptable quality. The mentioned modified cheeses were not significantly different (*p* > 0.05) from the cheese samples from the control group (they contained about 25% and 50% less Na, respectively). The ratio of Na:K in cheese salted with a mixture of NaCl/KCl in the ratio 1:1 was close to the desirable ratio recommended by nutritionists [43].

### 3.3. Microstructures of Modified WSBC

The SEM micrographs of WSBC with a reduced content of NaCl and a sodium–potassium salt combination used as a salt replacer are depicted in Figure 1. All the micrographs show a rough structure with small voids, and the replacement of NaCl with COMB resulted in obtaining a slightly more compact cheese structure. The differences in the microstructure of the cheese samples observed in the micrographs are due to changes in the protein gel network and can be correlated with different chemical and textural parameters of the cheese samples [93]. Although some differences among the samples were noticed, visually they cannot be considered to be of significant importance, as previously stated for most characteristics of the tested samples.

A similar structure with no significant difference in microstructure induced by the increase in KCl ratio was observed for white brine cheese in Jordan, Nabulsi cheese [76], and Halloumi cheese kept in various NaCl and KCl mixtures [78]. The most compact structure was observed for the cheese with 100% replacement of table salt. This sample of cheese also had smaller pores than the other cheese samples, which can indicate a stronger interaction between the particles. The reduction in pore diameter was observed with a higher potassium content, implying that the influence of K is on the level of interactions between the particles. However, an almost identical microstructure was observed between the samples PL1 and PL2, which indicated no difference in microstructure (replacement of 25% and 50% NaCl).

A large amount of KCl was proven to lead to a crumbly body of cheese [94], which can explain the denser structure of the sample PL4. The maximal concentration of KCl in the sample PL4 resulted in a significantly lower water content than in the control and, accordingly, a firmer cheese body. A slight increase in hardness with the addition of up to 1:1 NaCl:KCl was also reported for Nabulsi cheese [75]. Moreover, the investigations of the addition of whey salt (Na:K = 1:3.4) indicated that the increase in the amount of K resulted in the increased hardness and fracturability of white salted cheese [86]. This effect can be explained by the modification of protein hydration [86,95].

### 3.4. Sensory Acceptance of Modified Na-Reduced WSBC

The sensory acceptance scores related to the color, texture, odour, taste, and overall acceptance of the tested WSBC samples determined by a five-member panel of trained assessors are shown in Table 4.

The color of five tested control/modified WSBC samples was characteristically white and was rated as extremely acceptable, with scores ranging from 4.60 (PL-3) to 4.80 (PL-2, PL-4). The assessors did not observe any significant difference between the control group (CON) and the experimental lots (production lots PL-1 to PL-4). The reduction in NaCl did not have a significant effect on oral texture, and all five treatments were found to have very acceptable oral textures. However, the scores tended to slightly decrease with an increase in sodium substitution, from 4.40 for the control group to 3.70 for the PL-3 and PL-4 groups. Odor was evaluated as being very to extremely acceptable, with scores ranging from 4.00 for PL-4 samples to 4.50 for the CON group. Nevertheless, no significant difference was revealed between treatments. The taste score of PL-4 WSBC was significantly lower (*p* < 0.05) than for CON samples, but no statistically significant difference was observed in relation to the other groups of WSBC. The lower taste score for PL-4 cheese (3.30) was due to the presence of a bitter taste and reduced saltiness. A significant difference (*p* < 0.05) was noticed in overall acceptance between CON and PL-4 samples, with no statistically significant difference found between the other groups of WSBC. The samples belonging to PL-4 scored the worst in the sensory evaluation of the modified WSBC. In PL-4 samples, the Na content is close to the value that would allow it to be declared as “low-Na” in accordance with the legally prescribed nutritional statement.

According to Bansal and Mishra [69], replacing 25% to 50% of NaCl with KCl in Minas Padrão cheese did not result in any statistically significant differences in sensory acceptance. However, the replacement of more than 50% of NaCl with KCl, had a negative influence on the sensory properties of WSBC. The substitution above this percentage can cause cheeses to develop unpleasant tastes, such as bitter, acrid, chemical, and metallic tastes, which deter consumers from consuming such products. Nevertheless, KCl remains the most commonly used replacement of NaCl in the manufacture of low-Na WSBC due to their similar chemical structures [96]. Authors [68,73] revealed significant differences in aroma, flavor, texture, and overall acceptance with 70% salt substitution treatment (30% NaCl: 70% KCl). However, with 50% salt substitution, no significant differences were determined in aroma, texture, and overall acceptance. During the sensory evaluation, cheeses in which three-quarters of NaCl was replaced by KCl during salting were considered moderately to extremely acceptable by about 80% of consumers [18].

The results of oral texture and overall acceptance were consistent with the results of microstructure analysis. The sample with the highest reduction of NaCl (PL-4) had the most compact structure and smaller pores. This can explain why the scores for oral texture slightly decreased with an increase in Na substitution, from 4.40 for the CON to 3.70 for the PL-4 samples. In addition, a significant difference (*p* < 0.05) was noticed in overall acceptance between the CON and the PL-4 samples (4.30 and 3.50, respectively).

## 4. Conclusions

In order to reduce Na intake in consumers consuming WSBC, a mixture, i.e., a combination of Na and K mineral salts (COMB), was tested as a salt (NaCl) substitute added at four different concentrations. Physical-chemical analyses of modified WSBC samples were performed and the levels of essential minerals (main elements: Na, K, Mg, Ca, P, and microelements: Fe, Cu, Zn) were determined. In addition, their microstructures were analyzed using the SEM technique. Sensory evaluation of the finished products was performed by an expert panel. The replacement of table salt with COMB up to 75% did not significantly affect the quality of the modified WSBC. The results obtained in this research show that even 100% replacement of NaCl with COMB was not enough to label the modified cheese as “low-Na” cheese. In modified cheese samples from the experimental group PL-4, Na content was 334.80 ± 24.60 mg/100 g of Na. The FDA requires 280 mg/100 g of Na for “low-Na” cheeses. The Serbian Rulebook on Nutrition and Health Claims Labeled on Food Declarations stipulates the limit value of 120 mg/100 g Na for Na content in WSBC after 14 d of ripening. The results obtained in this study represent a good basis for the development of low-Na cheese production in Serbia. Good results from sensory analysis and a responsible approach to the health of all age categories and specific groups of people open new market segments for such production. Nevertheless, in order to implement WSBC production into the Serbian cheese industry, further investigation regarding the impact of low Na content on the microbial stability and shelf-life of cheese must be performed.

## Figures and Tables

**Table 1:** Ratio of NaCl content to the Na/K combination during salting of the CON and four PL of modified WSBC.

Experimental Groups	Dry Salting (Sprinkling)	Brine
CON	2% NaCl − 100 g/5 kg	1% NaCl − 50 g/5 kg
PL-1	25 g COMB + 75 g NaCl	12.5 g COMB + 37.5 g NaCl
PL-2	50 g COMB + 50 g NaCl	25 g COMB + 25 g NaCl
PL-3	75 g COMB + 25 g NaCl	37.5 g COMB + 12.5 g NaCl
PL-4	100 g COMB	50 g COMB

**Table 2:** Proximate composition, pH, and a_w_ values (mean values ± SD) of Serbian white soft-brined cheese (WSBC) with a reduced Na content.

Cheese Code	Moisture (%)	Dry Matter (%)	Protein	Fat	Ash	Titratable Acidity (°SH)	NaCl	pH	a_w_
			(%)	(%)	(%)		(%)		
CON	70.74 ± 0.28 ^a^	29.26 ± 0.28 ^c^	12.95 ± 0.08 ^c^	8.83 ± 0.26 ^a^	3.45 ± 0.02 ^a^	49.30 ± 0.17 ^b^	2.26 ± 0.07 ^a^	4.74 ± 0.28 ^a^	0.956 ± 0.002 ^a^
PL-1	69.43 ± 0.36 ^bc^	30.57 ± 0.36 ^abc^	12.71 ± 0.17 ^d^	8.17 ± 0.26 ^b^	3.19 ± 0.07 ^a^	55.70 ± 0.05 ^a^	1.83 ± 0.07 ^b^	4.63 ± 0.21 ^a^	0.956 ± 0.002 ^a^
PL-2	69.82 ± 0.43 ^ab^	30.18 ± 0.43 ^bc^	14.10 ± 0.16 ^a^	8.17 ± 0.26 ^b^	3.13 ± 0.13 ^a^	46.80 ± 0.06 ^c^	1.56 ± 0.07 ^c^	4.73 ± 0.21 ^a^	0.951 ± 0.003 ^a^
PL-3	68.58 ± 0.54 ^c^	31.42 ± 0.54 ^a^	13.70 ± 0.10 ^b^	8.58 ± 0.38 ^ab^	3.38 ± 0.18 ^a^	46.90 ± 0.05 ^bc^	1.33 ± 0.07 ^d^	4.64 ± 0.14 ^a^	0.956 ± 0.004 ^a^
PL-4	69.02 ± 0.17 ^bc^	30.98 ± 0.17 ^ab^	14.11 ± 0.14 ^a^	8.83 ± 0.26 ^a^	3.23 ± 0.18 ^a^	49.10 ± 0.09 ^bc^	0.94 ± 0.12 ^e^	4.56 ± 0.12 ^a^	0.949 ± 0.001 ^a^
*p* value	<0.001	<0.001	<0.001	<0.001	>0.05	<0.001	<0.001	>0.05	>0.05

^a–e^ Values (mean ± SD) in the same column with different letters are significantly different (*p* < 0.05). Cheese code: CON—cheese with 100% NaCl and 0% added Na/K salt combination (COMB); PL-1—cheese with 75% NaCl and 25% COMB; PL-2—cheese with 50%NaCl and 50% COMB; PL-3—cheese with 25%NaCl and 75% COMB; PL-4—cheese with 0% NaCl and 100% COMB.

**Table 3:** Contents of major- and micro-elements (mean values ± SD) in Serbian white soft-brined cheese (WSBC) with a reduced Na content.

Cheese Code	Na (mg/100 g)	K	Mg (mg/100 g)	Ca (mg/100 g)	P	Fe	Cu (mg/100 g)	Zn (mg/100 g)
		(mg/100 g)			(mg/100 g)	(mg/100 g)		
CON	777.40 ± 22.65 ^a^	131.56 ± 9.93 ^e^	15.70 ± 0.44 ^a^	498.06 ± 7.10 ^cd^	260.90 ± 1.60 ^bc^	1.11 ± 0.14 ^a^	0.26 ± 0.01^ab^	1.74 ± 0.17 ^a^
PL-1	402.65 ± 11.11 ^b^	162.86 ± 2.00 ^d^	15.48 ± 0.23 ^a^	475.08 ± 6.32 ^d^	255.80 ± 1.21 ^c^	1.03 ± 0.10 ^a^	0.30 ± 0.02 ^a^	1.22 ± 0.05 ^b^
PL-2	387.57 ± 17.67 ^b^	201.36 ± 4.89 ^c^	16.32 ± 0.02 ^a^	541.96 ± 3.97 ^a^	288.78 ± 0.26 ^a^	1.04 ± 0.17 ^a^	0.29 ± 0.03 ^a^	1.45 ± 0.15 ^ab^
PL-3	384.88 ± 10.77 ^b^	255.51 ± 9.01 ^b^	16.57 ± 0.76 ^a^	537.80 ± 20.70 ^ab^	289.67 ± 12.59 ^a^	1.10 ± 0.07 ^a^	0.31 ± 0.01 ^a^	1.60 ± 0.18 ^ab^
PL-4	334.83 ± 6.50 ^c^	298.43 ± 9.62 ^a^	16.45 ± 0.30 ^a^	511.38 ± 0.54 ^bc^	276.09 ± 0.95 ^ab^	0.79 ± 0.20 ^a^	0.21 ± 0.02 ^b^	1.78 ± 0.22 ^a^
*p* value	<0.001	<0.001	>0.005	<0.001	<0.001	>0.005	<0.05	<0.05

^a–e^ Values (mean ± SD) in the same column with different letters are significantly different (*p <* 0.05). Cheese code: same as for Table 2.

**Table 4:** Sensory acceptance scores (means ± standard deviation) of Serbian WSBC with a reduced Na content.

Cheese Code	Colour	Texture	Odour	Taste	Overall Acceptance
CON	4.70 ± 0.27 ^a^	4.40 ± 0.22 ^a^	4.50 ± 0.35 ^a^	4.40 ± 0.42 ^a^	4.30 ± 0.27 ^a^
PL-1	4.70 ± 0.27 ^a^	3.90 ± 0.55 ^a^	4.40 ± 0.42 ^a^	4.20 ± 0.27 ^a^	4.20 ± 0.27 ^a^
PL-2	4.80 ± 0.27 ^a^	3.80 ± 0.45 ^a^	4.30 ± 0.27 ^a^	3.90 ± 0.65 ^a^	4.00 ± 0.35 ^a^
PL-3	4.60± 0.42 ^a^	3.70 ± 0.57 ^a^	4.20 ± 0.27 ^a^	3.50 ± 0.87 ^a^	3.60 ± 0.55 ^a^
PL-4	4.80 ± 0.27 ^a^	3.70 ± 0.45 ^a^	4.00 ± 0.00 ^a^	3.30 ± 0.27 ^b^	3.50 ± 0.35 ^b^

^a,b^ Different superscript letters within a column indicate significant differences (*p* < 0.05); lack of superscripts indicates no significant differences (*p* > 0.05). Cheese code: CON—cheese with 100% NaCl and 0% added Na/K salt combination (COMB); PL-1—cheese with 75% NaCl and 25% COMB; PL-2—cheese with 50% NaCl and 50% COMB; PL-3—cheese with 25% NaCl and 75% COMB; PL-4—cheese with 0% NaCl and 100% COMB.

## Data Availability

The original contributions presented in the study are included in the article, further inquiries can be directed to the corresponding author.
